# Understanding Emotions Impacted by New Assessment Mandates Implemented in Medical Education: A Survey of Residents and Faculty Across Multiple Specialties

**DOI:** 10.7759/cureus.62013

**Published:** 2024-06-09

**Authors:** Sonaina Chopra, Jason M Harley, Amy Keuhl, Ereny Bassilious, Jonathan Sherbino, Elif Bilgic

**Affiliations:** 1 McMaster Education Research, Innovation and Theory (MERIT) Program, McMaster University, Hamilton, CAN; 2 Surgery, McGill University, Montreal, CAN; 3 Pediatric Endocrinology, McMaster University, Hamilton, CAN; 4 Emergency Medicine, McMaster University, Hamilton, CAN; 5 Pediatrics, McMaster University, Hamilton, CAN

**Keywords:** general surgery, emergency medicine, pediatrics, postgraduate medical education, entrustable professional activity, assessment, emotions

## Abstract

Background

Previous research findings show that the overall perception of residents regarding the new entrustable professional activity (EPA) assessment mandates is primarily negative. Hence, this study aims to explore the link between EPA assessment experiences and resident and faculty emotions and expectancy of successfully completing residency training.

Methods

A standardized questionnaire (Medical Emotions Scale (MES)), which measures 20 unique emotions on a 5-point Likert scale, was used to explore the emotions of residents and faculty members regarding EPA assessments and residents’ expectancy of success. Data analysis included descriptive statistics and analysis of variance (ANOVA).

Results

Ninety-one (N=91) participants (46 faculty members and 45 residents) completed the survey. The results revealed that residents have more negative emotions toward EPA assessments compared to faculty. Additionally, resident and faculty emotions regarding EPA assessments vary across specialty and gender.

Conclusions

These findings will be crucial in providing the Royal College of Physicians and Surgeons of Canada and medical education programs with concrete evidence and guidance in understanding the perspectives and emotions of residents and faculty towards EPA assessments and residents’ beliefs about successfully completing their medical training.

## Introduction

Medical education in Canada is currently shifting from the traditional apprenticeship model to an outcomes-based competence by design (CBD) model that focuses on skill development through specific learning objectives and better assessment strategies [1-3]. This new assessment model involves entrustable professional activities (EPAs), which are “essential tasks of a discipline that an individual can be entrusted to perform safely and independently”, that are assessed to ensure that, by the end of training, a trainee is ready for independent practice [1,4,5].

The transition to CBD and implementation of the new EPA assessment mandates are being done in increments [6]. Initial reports by the Federation of Quebec Medical Residents (FMRQ) and yearly snapshots by the Royal College of Physicians and Surgeons of Canada (RC) focusing on the experiences of trainees and faculty across programs and institutions suggest that the implementation of EPA assessments has caused negative emotions (e.g., anxiety, frustration, worry) in both trainees and faculty and uncertainty regarding success in medical training among trainees [7-9]. Some of the reasons included logistical difficulties (e.g., the assessment platform not functioning efficiently) and doubts regarding the educational benefit of CBD and EPA assessments.

The purpose of our study is to explore the emotions of faculty and residents regarding EPA assessments in three programs (pediatrics, emergency medicine, and general surgery) that implemented EPA assessments in recent years.

This article was previously presented as a meeting abstract at the 2023 Canadian Conference for the Advancement of Surgical Education on October 12, 2023.

## Materials and methods

The Strengthening the Reporting of Observational Studies in Epidemiology (STROBE) Statement for cross-sectional studies was used to guide the reporting.

Study Design

This was a cross-sectional observational study using surveys for data collection.

Conceptual Foundations

Positive emotions (e.g., hope, enjoyment) have been shown to have an overall positive impact on performance, which is the opposite of negative emotions (e.g., anxiety, hopelessness, frustration) [10]. We used Pekrun’s control-value theory (CVT) of achievement emotions to support our interpretation [11].

Setting, Participants, and Sampling

Through purposeful sampling, residents of all levels and faculty at McMaster University from general surgery, emergency medicine, and pediatrics, with prior experience completing and/or receiving EPA assessments, were recruited.

Data Sources/Measurement

Two surveys (see Appendix) were created in LimeSurvey (one for faculty, one for residents), and participants were provided with a $10 gift card honorarium. The surveys were piloted to ensure clarity and ease of completion, and the study was done from fall 2022 to winter 2023.

Both surveys included the Medical Emotion Scale (MES), which is a self-reporting standardized questionnaire that measures the intensity of 20 unique emotions on a 5-point Likert scale [12]. The MES categorizes emotions into four groups:

(i) Positive activating emotions (hopeful, proud, happy, enjoyment, compassionate, curious, grateful)

(ii) Positive deactivating emotions (relieved, relaxed)

(iii) Negative activating emotions (confused, angry, frustrated, afraid, anxious, ashamed)

(iv) Negative deactivating emotions (hopeless, disappointed, sad, bored)

Faculty and residents completed the MES to measure their emotions regarding EPA assessments. Residents also completed the MES to measure their emotions about successfully completing their residency training with EPA assessments. In our study, the MES demonstrated good scale reliability with Cronbach’s alpha ranging from 0.73 to 0.79. The survey also included questions with a textbox option to list the reasons for their ratings. Participants also provided their year of training/practice, specialty, gender, and ethnicity.

Ethical Consideration

This study received ethics approval from the Hamilton Integrated Research Ethics Board (HiREB #14686, August 2022), and consent forms were included on the first page of the surveys.

Statistical Methods

SPSS Statistics software was used for the analysis. Descriptive analyses (mean (M) and standard deviation (SD)) were performed. Next, a series of ANOVAs were performed to determine any significant differences in emotions of faculty and residents (and expectancy of success of residents) based on specialty (general surgery, emergency medicine, pediatrics), gender (man, woman, non-binary), ethnicity (minority vs non-minority), and the number of EPA assessments completed/received in past year (statistically significant, p <0.05). Effect size (Cohen’s d) was calculated to determine the strength of the significance [13]. Open-text box responses were analyzed using simple inductive thematic analysis [14].

Funding

This work is supported in part by funding from the Social Sciences and Humanities Research Council of Canada.

## Results

Forty-five residents (out of 104, a response rate of 43%) and 46 faculty (out of around 284 possible, a response rate of 16%) participated (details in Table 1). Compared to residents, faculty reported higher levels of positive activating (M=2.38, SD=0.93 vs M=2.07, SD=0.76) and positive deactivating (M=2.25, SD=1.01 vs M=1.97, SD=0.74) emotions.

**Table 1:** Summary of the demographic information (gender, specialty, minority affiliations, number of EPA assessments completed/received in the past year) of faculty and residents EPA: Entrustable Professional Activity The data have been represented as the number of participants (n) and percentage (%). *1 faculty and 1 resident selected 'I prefer not to answer'; hence, percentages for 'gender' do not add up to 100%.

Group (n)	Specialty (n (%))		Gender* (n (%))		Minority affiliations (n (%))		# of EPAs completed/received (n (%))	
Faculty (46)	General surgery	11 (24%)	Man	21 (46%)	Minority	12 (26%)	1-20 EPAs	14 (30%)
	Pediatrics	18 (39%)	Woman	24 (52%)	Non-minority	34 (74%)	21-50 EPAs	15 (33%)
	Emergency medicine	17 (37%)	Non-binary	0 (0%)	-	-	51+	17 (37%)
Residents (45)	General surgery	16 (36%)	Man	11 (24%)	Minority	17 (38%)	1-30 EPAs	12 (27%)
	Pediatrics	19 (42%)	Woman	33 (73)	Non-minority	28 (62%)	31-50 EPAs	19 (42%)
	Emergency medicine	10 (22%)	Non-binary	0 (0%)	-	-	51+	14 (31%)

Faculty (Completing EPA Assessments)

Faculty demonstrated higher levels of positive activating (e.g., happy) (M=2.38, SD=0.93) and positive deactivating emotions (e.g., relief) (M=2.25, SD=1.01) compared to negative emotions (e.g., angry, disappointed). The findings of the factorial ANOVA indicated that there are no statistically significant variations in faculty emotions based on specialty, gender, minority group affiliation, or the number of completed EPA assessments.

Based on the text responses, faculty reported challenges, including the perception that EPA assessments are reasonable for most residents but can be stressful and fatiguing for individuals needing remediation. Additionally, there were concerns about residents selectively engaging EPA assessments when they predicted to perform well.

Residents (Receiving EPA Assessments and Expectancy of Success)

Residents demonstrated higher levels of negative activating (e.g., angry) (M=2.81, SD=0.96) and negative deactivating emotions (e.g., disappointed) (M=2.77, SD=0.97) compared to positive emotions (e.g., happy, relieved).

Factorial ANOVA results were similar between the two MES completed by residents (details in Table 2). First, for differences across specialties (general surgery, pediatrics, and emergency medicine), a statistically significant difference was observed in positive deactivation emotions (relieved, relaxed). Specifically, residents from general surgery reported higher levels and residents from emergency medicine reported lower levels: F (2, 42)=3.35, p<0.05, with an effect size of 0.138.

**Table 2:** Summary of the descriptive analysis and ANOVAs for resident emotions regarding receiving EPA assessments based on specialty, gender, minority affiliations, and number of EPA assessments received in the past year The data have been represented as N, %, mean±SD, F, and significance. **P<0.05, ***P<0.001 NS: Not Significant This table includes the descriptive (mean and standard deviation) and a between-group ANOVA of four categories of emotions from the Medical Emotions Scale (MES) to analyze if there is a significant difference in residents’ emotions based on specialty (emergency medicine, pediatrics, general surgery), gender (man, woman, non-binary) minority affiliation, and the number of EPA assessments received in the past year (1-30, 31-50, 51). The MES measures the range of 20 unique emotions on a 5-point Likert scale. The emotions are categorized into four groups: (i) positive activating emotions (hopeful, proud, happy, enjoyment, compassionate, curious, grateful), (ii) positive deactivating emotions (relieved, relaxed), (iii) negative activating emotions (confused, angry, frustrated, afraid, anxious, ashamed), and (iv) negative deactivating emotions (hopeless, disappointed, sad, bored).

Emotions	Specialty (n (%))	Mean	SD	F	Sig
Positive activating	Emergency medicine (10 (22%))	1.91	0.522	0.858	NS
	Pediatrics (19 (42%))	1.92	0.786	-	-
	General surgery (16 (36%))	2.23	0.851	-	-
Positive deactivating	Emergency medicine (10 (22%))	2.20	0.483	3.35	**
	Pediatrics (19 (42%))	1.66	0.625	-	-
	General surgery (16 (36%))	2.22	0.894	-	-
Negative activating	Emergency medicine (10 (22%))	2.45	0.835	2.28	NS
	Pediatrics (19 (42%))	3.14	0.944	-	-
	General surgery (16 (36%))	2.62	0.990	-	-
Negative deactivating	Emergency medicine (10 (22%))	2.52	0.803	0.853	NS
	Pediatrics (19 (42%))	2.99	0.970	-	-
	General surgery (16 (36%))	2.67	1.09	-	-
Emotions	Gender (n (%))	Mean	SD	F	Sig
Positive activating	Woman (33 (75%))	1.92	0.730	3.27	**
	Man (11 (25%))	2.45	0.7022	-	-
Positive deactivating	Woman (33 (75%))	1.85	0.655	4.11	**
	Man (11 (25%))	2.45	0.820	-	-
Negative activating	Woman (33 (75%))	2.84	0.964	1.86	NS
	Man (11 (25%))	2.86	0.893	-	-
Negative deactivating	Woman (33 (75%))	2.79	0.893	1.78	NS
	Man (11 (25%))	2.89	1.16	-	-
Emotions	Minority (n (%))	Mean	SD	F	Sig
Positive activating	Yes (17 (39%))	0.669	1.05	0.994	NS
	No (27 (61%))	2.08	0.811	-	-
Positive deactivating	Yes (17 (39%))	2.15	0.825	1.45	NS
	No (27 (61%))	1.91	0.678	-	-
Negative activating	Yes (17 (39%))	2.89	0.983	1.90	NS
	No (27 (61%))	2.82	0.924	-	-
Negative deactivating	Yes (17 (39%))	2.76	1.032	1.77	NS
	No (27 (61%))	2.84	0.918	-	-
Emotions	# of EPAs in the past year (n (%))	Mean	S. D	F	Sig
Positive activating	1-30 (12 (27%))	2.02	0.76	0.062	NS
	31-50 (19 (42%))	2.0	0.74	-	-
	51+ (14 (31%))	2.09	0.841	-	-
Positive deactivating	1-30 (12 (27%))	2.25	0.89	1.17	NS
	31-50 (19 (42%))	1.92	0.61	-	-
	51+ (14 (31%))	1.82	0.77	-	-
Negative activating	1-30 (12 (27%))	2.67	0.87	0.194	NS
	31-50 (19 (42%))	2.82	0.89	-	-
	51+ (14 (31%))	2.91	1.12	-	-
Negative deactivating	1-30 (12 (27%))	2.67	0.944	0.286	NS
	31-50 (19 (42%))	2.88	0.96	-	-
	51+ (14 (31%))	2.76	1.07	-	-

Second, for differences across gender, there were statistically significant differences in positive activating emotions (e.g., hopeful, proud, happy) - residents who identify as man scored having higher levels than residents who identify as woman: F (2, 42)=3.27, p<0.05, with an effect size of 0.135. Similar statistical significance was found in positive deactivating emotions (relieved, relaxed) - residents who identify as man scored having higher levels than residents who identify as woman: F (2, 42)=4.11, p<0.05.

Finally, there were no statistically significant differences in residents’ emotions based on minority group affiliation or the number of EPA assessments received.

Residents’ Written Responses

Some challenges expressed included receiving minimal and non-useful feedback, encountering inconsistent grading practices, perceiving EPA assessments as a checkbox rather than a valuable learning tool, and experiencing frustration when faculty failed to complete EPA assessments in a timely manner.

## Discussion

In Canada, emergency medicine, general surgery, and pediatrics transitioned to CBD in 2018, 2020, and 2021, respectively [6]. Both faculty and residents in general surgery reported higher positive emotions related to EPA assessments compared to emergency medicine and pediatrics. First, emergency medicine was one of the first programs to transition to CBD in 2018 [6]. Therefore, we could hypothesize that faculty and trainees feel more negative emotions toward EPA assessments due to more time potentially struggling with implementing CBD. Pediatrics transitioned to CBD in 2021, so perhaps their negative emotions toward EPA assessments are due to the challenges that come with a more recent transition to CBD. The stronger negative emotions observed in emergency medicine and pediatrics could also be due to differences in how CBD/EPA assessments are being implemented in each specialty, including different strategies used by programs to overcome challenges.

In our resident sample, a notable trend was that residents who identify as man exhibited significantly higher levels of positive activation (happy, hopeful) and positive deactivation emotions (relieved, relaxed) compared to residents who identify as woman. This was not observed in our faculty sample. Existing research suggests that it is pertinent to consider the impact of gender biases in medical education. For example, female residents are more likely to receive lower ratings and less positive feedback on their performance assessments [15]. This discrepancy in feedback could potentially influence female residents' emotions towards their EPA assessments negatively. In another study on EPA assessments, females rated their own performances lower than their male counterparts, despite faculty assessments not showing any differences between female and male resident performance [16]. Therefore, our observed gender differences in emotions toward EPA assessments may be influenced by a combination of factors associated with self-perception and potential biases in the assessment process.

In general, residents exhibited predominantly negative emotions towards EPA assessments and expectancy of success. Therefore, it is imperative for residency programs to gain a comprehensive understanding of the emotional experiences of residents and faculty toward CBD implementation. In doing so, they can proactively undertake initiatives aimed at addressing negative emotions and implementing improvements that have the potential to positively influence residents' emotions, thereby contributing to the overall success of the residents.

For this study, self-reporting questionnaires were used instead of other methods of measuring emotions. The MES relies on participants' interpretations and their individual manner of reporting their emotions. While there are other various methods to measure emotions, including examining brain activity, salivary biomarker tests, skin conductance, tracking eye movements, and analyzing facial expressions and observable responses, self-reporting measures remain one of the most widely utilized methods for data collection to measure emotions [12,17,18]. Specifically for emotions, self-reporting measures possess the capability to capture a broad range of emotions and demonstrate higher efficiency [19]. Additionally, self-report questionnaires offer advantages in terms of time and cost-effectiveness [20]. Given that emotions have a subjective experiential component, they are effectively measured through self-reported measures [12].

For limitations, first, the study was conducted at a single institution, which may restrict the generalizability. Second, for faculty, due to how the listservs are organized, it was hard to determine the exact response rate. Finally, conducting qualitative investigations would help with gaining a deeper understanding of the underlying factors influencing faculty and resident emotions and the expectancy of success related to EPA assessments.

## Conclusions

To summarize, this study performed an exploration of the link between EPA assessments and resident/faculty emotions and expectancy of success. Our findings suggest that, overall, residents have more negative emotions associated with EPA assessments compared to faculty that vary across specialty and gender. Therefore, investigations such as interviews could help further understand why residents and faculty feel a certain way to then develop concrete action plans.

## Appendices

**Table 3:** Survey of faculty and residents

Faculty Survey	
Question Group 1: Eligibility	
1. Have you completed an Entrustable Professional Activity (EPA) assessment for one or more residents in the past year?	
· Yes [show next question]	
· No [Unfortunately, you are not eligible to take part in this survey. Please close the survey.]	
2. What is your specialty?	
· Emergency Medicine [continue]	
· Pediatrics [continue]	
· General Surgery [continue]	
· Other [Unfortunately, you are not eligible to take part in this survey. Please close the survey.]	
Question Group 2: Emotions	
Using the scales below, indicate how you currently feel about completing EPA assessments. For each emotion, please select the number that best describes the intensity of your emotions.	
1. Confused	
(1) Not at all, (2) Very little, (3) Moderate, (4) Strong, (5) Very Strong	
2. Bored	
(1) Not at all, (2) Very little, (3) Moderate, (4) Strong, (5) Very Strong	
3. Hopeful	
(1) Not at all, (2) Very little, (3) Moderate, (4) Strong, (5) Very Strong	
4 Proud	
(1) Not at all, (2) Very little, (3) Moderate, (4) Strong, (5) Very Strong	
5. Sad	
(1) Not at all, (2) Very little, (3) Moderate, (4) Strong, (5) Very Strong	
6. Anxious	
(1) Not at all, (2) Very little, (3) Moderate, (4) Strong, (5) Very Strong	
7. Frustrated	
(1) Not at all, (2) Very little, (3) Moderate, (4) Strong, (5) Very Strong	
8. Happy	
(1) Not at all, (2) Very little, (3) Moderate, (4) Strong, (5) Very Strong	
9. Hopeless	
(1) Not at all, (2) Very little, (3) Moderate, (4) Strong, (5) Very Strong	
10. Enjoyment	
(1) Not at all, (2) Very little, (3) Moderate, (4) Strong, (5) Very Strong	
11. Ashamed	
(1) Not at all, (2) Very little, (3) Moderate, (4) Strong, (5) Very Strong	
12. Compassionate	
(1) Not at all, (2) Very little, (3) Moderate, (4) Strong, (5) Very Strong	
13. Surprised	
(1) Not at all, (2) Very little, (3) Moderate, (4) Strong, (5) Very Strong	
14. Curious	
(1) Not at all, (2) Very little, (3) Moderate, (4) Strong, (5) Very Strong	
15. Afraid	
(1) Not at all, (2) Very little, (3) Moderate, (4) Strong, (5) Very Strong	
16. Grateful	
(1) Not at all, (2) Very little, (3) Moderate, (4) Strong, (5) Very Strong	
17. Disappointed	
(1) Not at all, (2) Very little, (3) Moderate, (4) Strong, (5) Very Strong	
18. Relieved	
(1) Not at all, (2) Very little, (3) Moderate, (4) Strong, (5) Very Strong	
19. Angry	
(1) Not at all, (2) Very little, (3) Moderate, (4) Strong, (5) Very Strong	
20. Relaxed	
(1) Not at all, (2) Very little, (3) Moderate, (4) Strong, (5) Very Strong	
21. Please select the extent to which you agree/disagree with the following sentence:	
I feel in control of my performance as a supervising faculty when completing EPA assessments.	
Strongly disagree 1 2 3 4 5 Strongly agree	
22. Please select the extent to which you agree/disagree with the following sentence.	
I value completing EPA assessments.	
Strongly disagree 1 2 3 4 5 Strongly agree	
23. In the space below, please explain the factors that may have contributed to your answers above, regarding your current feelings about completing EPA assessments.	
[large textbox]	
Question Group 3: Demographics	
1. What is your gender?	
· Man	
· Woman	
· Non-binary/other	
· I prefer not to answer	
2. How many years have you been in practice?	
· 0-1 year	
· 2-5 years	
· 6-10 years	
· 11-15 years	
· 16+ years	
3. Do you identify as a member of a visible minority in Canada?	
· Yes	
· No	
· I prefer not to answer	
3a. If yes, please select the option(s) that you identify with.	
[checkboxes]	
· Arab	
· Black	
· Chinese	
· Filipino	
· Japanese	
· Korean	
· South Asian (e.g; East Indian, Pakistani, Sri Lankan)	
· Southeast Asian (e.g; Vietnamese, Cambodian, Laotian, Thai, etc)	
· West Asian (e.g; Iranian, Afgan, etc)	
· Other [short answer text box]	
4. Do you identify as Indigenous, meaning First Nations (North American Indian), Metis, or Inuit?	
· Yes	
· No	
· Prefer not to answer	
5. Do you have a learning disability or medical condition that affects your ability to complete EPA assessments?	
· Yes	
· No	
· Prefer not to answer	
6. In what year did you start completing EPA assessments? [number text box]	
7. Estimate the number of EPA assessments that you have completed in the past year (If you started your practice less than a year ago, please consider since the start of your faculty position):	
· 10-20	
· 21-50	
· 51-100	
· 101-200	
· 201+	
8. Estimate the number of EPA assessments that you have completed in total (include all years of doing EPA assessments):	
· 1-20	
· 21-50	
· 51-100	
· 101-200	
· 201-300	
· 301-400	
· 401+	
Survey for Residents	
Question Group 1: Eligibility	
1. Have you received an Entrustable Professional Activity (EPA) assessment in the past year?	
· Yes [Go to Specialty Question]	
· No [Unfortunately, you are not eligible to do this survey. Please close the survey.]	
2. What is your specialty?	
· Emergency Medicine [continue]	
· Pediatrics [continue]	
· General Surgery [continue]	
· Other [Unfortunately, you are not eligible to do this survey. Please close this survey.]	
Question Group 2: Emotions	
Using the scales below, please indicate how you currently feel about EPA assessments in general. For each emotion, please indicate the strength of that emotion by selecting the number that best describes the intensity of your emotion.	
1. Confused	
(1) Not at all, (2) Very little, (3) Moderate, (4) Strong, (5) Very Strong	
2. Bored	
(1) Not at all, (2) Very little, (3) Moderate, (4) Strong, (5) Very Strong	
3. Hopeful	
(1) Not at all, (2) Very little, (3) Moderate, (4) Strong, (5) Very Strong	
4. Proud	
(1) Not at all, (2) Very little, (3) Moderate, (4) Strong, (5) Very Strong	
5. Sad	
(1) Not at all, (2) Very little, (3) Moderate, (4) Strong, (5) Very Strong	
6. Anxious	
(1) Not at all, (2) Very little, (3) Moderate, (4) Strong, (5) Very Strong	
7. Frustrated	
(1) Not at all, (2) Very little, (3) Moderate, (4) Strong, (5) Very Strong	
8. Happy	
(1) Not at all, (2) Very little, (3) Moderate, (4) Strong, (5) Very Strong	
9. Hopeless	
(1) Not at all, (2) Very little, (3) Moderate, (4) Strong, (5) Very Strong	
10. Enjoyment	
(1) Not at all, (2) Very little, (3) Moderate, (4) Strong, (5) Very Strong	
11. Ashamed	
(1) Not at all, (2) Very little, (3) Moderate, (4) Strong, (5) Very Strong	
12. Compassionate	
(1) Not at all, (2) Very little, (3) Moderate, (4) Strong, (5) Very Strong	
13. Surprised	
(1) Not at all, (2) Very little, (3) Moderate, (4) Strong, (5) Very Strong	
14. Curious	
(1) Not at all, (2) Very little, (3) Moderate, (4) Strong, (5) Very Strong	
15. Afraid	
(1) Not at all, (2) Very little, (3) Moderate, (4) Strong, (5) Very Strong	
16. Grateful	
(1) Not at all, (2) Very little, (3) Moderate, (4) Strong, (5) Very Strong	
17. Disappointed	
(1) Not at all, (2) Very little, (3) Moderate, (4) Strong, (5) Very Strong	
18. Relieved	
(1) Not at all, (2) Very little, (3) Moderate, (4) Strong, (5) Very Strong	
19. Angry	
(1) Not at all, (2) Very little, (3) Moderate, (4) Strong, (5) Very Strong	
20. Relaxed	
(1) Not at all, (2) Very little, (3) Moderate, (4) Strong, (5) Very Strong	
21. Please select the extent to which you agree/disagree with the following sentence:	
I feel in control of my performance with EPA assessments.	
Strongly disagree 1 2 3 4 5 Strongly agree	
22. Please select the extent to which you agree/disagree with the following sentence:	
I value EPA assessments.	
Strongly disagree 1 2 3 4 5 Strongly agree	
23. In the space below, please explain the factors that may have contributed to your answers above, regarding your current feelings about EPA assessments.	
[large text box]	
Question Group 3	
Using the scale below, please indicate how you currently feel about your goal to successfully complete your residency training with the current usage of EPA assessments. For each emotion, please indicate the strength of that emotion by selecting the number that best describes the intensity of your emotion.	
1. Confused	
(1) Not at all, (2) Very little, (3) Moderate, (4) Strong, (5) Very Strong	
2. Bored	
(1) Not at all, (2) Very little, (3) Moderate, (4) Strong, (5) Very Strong	
3. Hopeful	
(1) Not at all, (2) Very little, (3) Moderate, (4) Strong, (5) Very Strong	
4. Proud	
(1) Not at all, (2) Very little, (3) Moderate, (4) Strong, (5) Very Strong	
5. Sad	
(1) Not at all, (2) Very little, (3) Moderate, (4) Strong, (5) Very Strong	
6. Anxious	
(1) Not at all, (2) Very little, (3) Moderate, (4) Strong, (5) Very Strong	
7. Frustrated	
(1) Not at all, (2) Very little, (3) Moderate, (4) Strong, (5) Very Strong	
8. Happy	
(1) Not at all, (2) Very little, (3) Moderate, (4) Strong, (5) Very Strong	
9. Hopeless	
(1) Not at all, (2) Very little, (3) Moderate, (4) Strong, (5) Very Strong	
10. Enjoyment	
(1) Not at all, (2) Very little, (3) Moderate, (4) Strong, (5) Very Strong	
11. Ashamed	
(1) Not at all, (2) Very little, (3) Moderate, (4) Strong, (5) Very Strong	
12. Compassionate	
(1) Not at all, (2) Very little, (3) Moderate, (4) Strong, (5) Very Strong	
13. Surprised	
(1) Not at all, (2) Very little, (3) Moderate, (4) Strong, (5) Very Strong	
14. Curious	
(1) Not at all, (2) Very little, (3) Moderate, (4) Strong, (5) Very Strong	
15. Afraid	
(1) Not at all, (2) Very little, (3) Moderate, (4) Strong, (5) Very Strong	
16. Grateful	
(1) Not at all, (2) Very little, (3) Moderate, (4) Strong, (5) Very Strong	
17. Disappointed	
(1) Not at all, (2) Very little, (3) Moderate, (4) Strong, (5) Very Strong	
18. Relieved	
(1) Not at all, (2) Very little, (3) Moderate, (4) Strong, (5) Very Strong	
19. Angry	
(1) Not at all, (2) Very little, (3) Moderate, (4) Strong, (5) Very Strong	
20. Relaxed	
(1) Not at all, (2) Very little, (3) Moderate, (4) Strong, (5) Very Strong	
21. Please select the extent to which you agree/disagree with the following sentence for your residency training with the usage of EPA assessments:	
I feel in control of my performance concerning my residency training with the usage of EPA assessments.	
Strongly disagree 1 2 3 4 5 Strongly agree	
22. Please select the extent to which you agree/disagree with the following sentence for your residency training with the usage of EPA assessments:	
I value the role of EPA assessments within my residency training.	
Strongly disagree 1 2 3 4 5 Strongly agree	
23. Please describe any factors that may have contributed to your answers above regarding your feelings about successfully completing your residency training with the current usage of EPA assessments. [not mandatory]	
[large text box]	
Question Group 3: Demographics	
1. What is your gender?	
· Man	
· Woman	
· Non-binary/other	
· I prefer not to answer	
2. What year of residency are you in?	
· Year 1	
· Year 2	
· Year 3	
· Year 4	
· Year 5	
3. Do you identify as a member of a visible minority in Canada?	
· Yes	
· No	
· I prefer not to answer	
3a. Please select the option(s) that you identify with. [checkboxes]	
[This question will only show if they select ‘yes’ in Q4 above]	
· Arab	
· Black	
· Chinese	
· Filipino	
· Japanese	
· Korean	
· South Asian (e.g; East Indian, Pakistani, Sri Lankan)	
· Southeast Asian (e.g; Vietnamese, Cambodian, Laotian, Thai, etc)	
· West Asian (e.g; Iranian, Afgan, etc)	
· Other [short text box]	
4. Do you identify as Indigenous, meaning First Nations (North American Indian), Metis, or Inuit?	
· Yes	
· No	
· Prefer not to answer	
5. Do you have a learning disability or medical condition that affects your ability to receive or complete EPA assessments?	
· Yes	
· No	
· Prefer not to answer	
6. Estimate the number of EPA assessments you have received/completed in the past year. If you started your residency less than a year ago, please consider since the start of residency program:	
· 1-10	
· 11-30	
· 31-50	
· 51-70	
· 71+	
7. Estimate the number of EPA assessments you have completed in total (for all your years of residency training):	
· 1-10	
· 11-30	
· 31-50	
· 51-70	
· 71-100	
· 101-150	
· 151+	
